# D-Galactose Induces Chronic Oxidative Stress and Alters Gut Microbiota in Weaned Piglets

**DOI:** 10.3389/fphys.2021.634283

**Published:** 2021-04-08

**Authors:** Hui Han, Zemin Liu, Jie Yin, Jing Gao, Liuqin He, Chenyu Wang, Ruoxin Hou, Xingguo He, Guoqiang Wang, Tiejun Li, Yulong Yin

**Affiliations:** ^1^Key Laboratory of Agro-ecological Processes in Subtropical Region, Hunan Provincial Key Laboratory of Animal Nutritional Physiology and Metabolic Process, National Engineering Laboratory for Pollution Control and Waste Utilization in Livestock and Poultry Production, Institute of Subtropical Agriculture, Chinese Academy of Sciences, Changsha, China; ^2^State Key Laboratory of Animal Nutrition, Institute of Animal Science, Chinese Academy of Agricultural Sciences, Beijing, China; ^3^College of Advanced Agricultural Sciences, University of Chinese Academy of Sciences, Beijing, China; ^4^College of Animal Science and Technology, Hunan Agricultural University, Changsha, China; ^5^Research Institute of Oil Tea Camellia, Hunan Academy of Forestry, Changsha, China; ^6^National Engineering Research Center for Oil Tea Camellia, Changsha, China; ^7^Hunan International Joint Laboratory of Animal Intestinal Ecology and Health, Laboratory of Animal Nutrition and Human Health, College of Life Sciences, Hunan Normal University, Changsha, China; ^8^Changsha Lvye Bio-Technology Co., Ltd., Changsha, China

**Keywords:** D-galactose, chronic oxidative stress, gut microbiota, weaned piglets, growth performance

## Abstract

Oxidative stress commonly occurs in pig production, which can severely damage the intestinal function of weaned piglets. This study was conducted to investigate the effects of D-galactose with different levels used to induce chronic oxidative stress on growth performance, intestinal morphology and gut microbiota in weaned piglets. The results showed that addition of 10 and 20 g/kg BW D-galactose reduced average daily gain and average daily feed intake from the first to the third week. 10 g/kg BW D-galactose increased the concentration of serum MDA at the second and third week. 10 g/kg BW D-galactose significantly influenced the jejunal and ileal expressions of *GPx1*, *CAT1*, and *MnSOD*. The results of 16S rRNA sequencing showed that compared with the control, 10 and 20 g/kg BW D-galactose significantly decreased the relative abundance of *Tenericutes*, *Erysipelotrichia*, *Erysipelotrichales*, and *Erysipelotrichaceae*, while increased the relative abundance of *Negativicutes*, *Selenomonnadales*, and *Veillonellaceae*. The results indicated that treatment with 10 g/kg BW/day D-galactose for 3 weeks could induce chronic oxidative stress, reduce the growth performance and alter gut microbiota in weaned piglets.

## Introduction

Oxidative stress is regarded as an imbalance between the oxidative and antioxidative reactions (Jiang et al., 2016; Yahata and Hamaoka, 2016). Under normal physiological circumstance, reactive oxygen species (ROS) are maintained homeostasis and excessive ROS are eliminated by the antioxidant system including non-enzymatic components and antioxidant enzymes, such as glutathione peroxidase (GSH-Px), catalase (CAT), and superoxide dismutase (SOD) (Jiang et al., 2016). However, the imbalance between the prooxidant and antioxidant activity will lead to the excess production of ROS, which can cause oxidative stress and do harm to cellular DNA, protein, and lipid (Ali et al., 2015; Jiang et al., 2016).

Post-weaning piglets are subjected to oxidative stress caused by the changes of nutritional source, physical and social environments (Yin et al., 2014). During weaning, piglets have to be suddenly separated from the sow, transported to a different physical environment, regrouped with unfamiliar piglets from other litters, changed from sow watery milk to feeding on solid feed, and exposed to increased dietary or environmental pathogens and antigens (Campbell et al., 2013). Amounting studies have shown that gastrointestinal tract is highly susceptible to oxidative stress and previous investigations have revealed that oxidative stress can damage the intestinal barrier functions and change the gut microbiome in piglets (He et al., 2017; Zheng et al., 2017; Cao et al., 2018; Zhang et al., 2018). Gut microbiota and intestinal barrier are strongly related to maintaining homeostasis in intestine as well as other organs of host (Dam et al., 2019). Disordered gut microbiota can produce excessive ROS to induce oxidative stress that in turn lead to intestinal inflammation and even various chronic diseases related to inflammation (Marciano and Vajro, 2017; Vasquez et al., 2019). However, some gut microbial metabolites, such as SCFAs, are able to alleviate oxidative stress (Andrade-Oliveira et al., 2015; Rose et al., 2018). Treatment with diquat and polyunsaturated fatty acids are always used to induce acute or chronic oxidative stress in piglets (Di Giancamillo et al., 2015; Shen et al., 2015; Cao et al., 2018; Rossi et al., 2019). However, there are several disadvantages to using diquat or polyunsaturated fatty acids as treatments. Firstly, intraperitoneal injection of diquat can cause severe stress in piglets, such as anorexia and diarrhea, which might affect the results of following study (Xu et al., 2018). Secondly, vegetable oil and fish oil enriched in polyunsaturated fatty acids are commonly used to induced chronic oxidative stress but the fresh oil need to be pre-oxidized and determined the peroxide value before supplementation, which is a little complex (Shan et al., 2009; Di Giancamillo et al., 2015; Wang et al., 2016).

To determine the mechanism whereby oxidative stress exerts effects on intestinal health and gut microbiota, the chronic oxidative stress is suitable for observing the change trend of piglets than acute oxidative stress. D-galactose, as a monosaccharide sugar, can be metabolized by mammalian animals. However, excess administration of D-galactose can induce oxidative stress in the body by three different ways. Firstly, D-galactose, as a reducing sugar, can react with the free amines of amino acids and produce a Schiff base (Shwe et al., 2018). The Schiff is oxidized and form advanced glycation end products, which can interact with specific receptors and then induce the production of reactive oxygen species (ROS) (Zhang et al., 2016). Excessive ROS cause excess peroxidation of proteins, lipid, and DNA. Secondly, high level of D-galactose can induce the production of hydrogen peroxide and MDA and inhibit the levels of antioxidant enzymes, such as SOD and GSH (Shwe et al., 2018; Zhang Z. et al., 2019). Thirdly, D-galactose is reduced by galactose reductase and over-supply D-galactose converts into galactitol, which cannot be metabolized and then accumulate in the cell to generate amounts of ROS (Thakur et al., 2017). It is well known that D-galactose treatment is widely used as a chronic model to induce oxidative stress in rats and mice (Shen et al., 2002; Qian et al., 2018; Zhang X. et al., 2019). However, there is currently no study on chronic oxidative stress induced by D-galactose in a pig model. Thus, our study was conducted to test whether D-galactose could be used as a model to induce chronic oxidative stress and investigate the effects of chronic oxidative stress on growth performance, intestinal morphology and microbiota in weaned piglets.

## Materials and Methods

### Animal and Experimental Design

This study was approved by the animal welfare committee of the Institute of Subtropical Agriculture, Chinese Academy of Sciences (2013020; Changsha, China). Thirty-two cross-bred (Duroc × Landrace × Yorkshire; average body weight (BW) = 5.44 ± 0.26 kg) healthy piglets weaned at 21 days were randomly divided into four treatments (*n* = 8/group): (1) control group, piglets were fed the basal diet; (2) 5 g/kg BW D-galactose group, piglets were fed with the basal diet supplemented with D-galactose at a dosage of 5 g/kg BW/day; (3) 10 g/kg BW D-galactose group, piglets were fed with the basal diet supplemented with D-galactose at a dosage of 10 g/kg BW/day; and (4) 20 g/kg BW D-galactose group, piglets were fed the basal diet supplemented with D-galactose at a dosage of 20 g/kg BW/day. The BW were measured weekly and the volume of D-galactose were calculated according to BW. Every morning, after cleaning the through and weighing the remaining feed, D-galactose was mixed in a small amount of diet and given to the piglets. Then the piglets were fed with diet without D-galactose until they eat up the diet containing D-galactose. The composition and nutrient levels of the basal diet met the nutrient specifications for 5 to 10 kg BW pig according to recommendations of the National Research Council (2012) (Table 1). During the whole experiment, piglets were housed individually and given free access to water. The average daily feed intake (ADFI) was monitored daily and ADFI and final average daily gain (ADG) were calculated weekly. The duration of the overall experiment was 21 days. The administration dosages of D-galactose (Hubei Yuying Biotechnology Co., Ltd., Yichang, Hubei, China) were adopted according to the previous mice experiments (Ma et al., 2018; Sun et al., 2018; Sha et al., 2019).

**TABLE 1:** Composition and nutrient level of basal diet.

Item	Composition %
Corn	55.59
Soybean oil	0.72
Glucose	1
Sucrose	2
Expanded soybean	8
Soybean meal	21.5
Fish meal	4
Whey power	3.2
NaCl	0.4
Limestone	0.55
CaHCO_3_	0.87
ZnO	0.3
Lysine	0.4
Methionine	0.2
Threonine	0.14
Tryptophan	0.03
Choline chloride	0.1
Premix*	1
Nutritional Level	
CP	19.74
Lysine	1.39
Methionine + Cysteine	0.84
Threonine	0.88
Tryptophan	0.24

*Premix provided the following per kilogram of the diet: Sepiolite, 6.043 g; pig vitamin, 750 mg; Fe, 150 mg; Cu, 150 mg; Man, 80 mg; Zn, 120 mg; Se, 0.3 mg; Co, 1 mg; I, 0.3 mg; VB4 1,000 mg.

### Sample Collection

Blood sample of the piglets in every week were collected and serum were obtained by centrifugation at 3,000 rpm for 10 min under 4°C and stored at −20°C until analysis according to our previous studies (Yin et al., 2017). On days 21, after blood sampling, all piglets were anesthetized with an intravenous injection of sodium pentobarbital (50 mg/kg BW) and bled by exsanguination. The small intestine was dissected free of the mesentery and sampled on a chilled stainless-steel tray. Ten-cm segments were collected from the proximal jejunum and distal ileum, respectively, thoroughly flushed with ice-cold phosphate-buffered saline, and then frozen in liquid nitrogen and stored at −80°C for the analysis of gene expression, one segment was fixed in 4% paraformaldehyde-PBS for examination of intestinal morphology. Colonic digesta was collected to determine gut microbiota. The heart, liver, spleen, and kidney were isolated and weighted. Relative organ weight (g/kg) = organ weight (g)/body weight (kg).

### Intestinal Histomorphology

Jejunal and ileal sections were fixed with 4% paraformaldehyde-PBS overnight, then dehydrated and embedded in paraffin blocks and sectioned at 5–7 μm. The sections were further deparaffinized and hydrated, and then stained with hematoxylin eosin (H&E). Villus length and crypt depth were performed using image J software.

### Serum Biochemical Parameters and Amino Acids Determination

Serum biochemical parameters, including total protein (TP), albumin (ALB), alanine transaminase (ALT), glutamic oxalacetic transaminase (AST), alkaline phosphatase (ALP), cholesterol (CHOL), high density lipoprotein (HDL), low density lipoprotein (LDL), were determined using automatic biochemical analyzer (fully automatic bio analysis machine cobas c311, Roche Co., Ltd.). Serum amino acids contents (His, Ser, Arg, Gly, Asp, Glu, Thr, Ala, Pro, Cys, Lys, Tyr, Met, Val, Ile, Leu, Phe, and Trp) were measured by High-speed Amino Acid Analyzer L-8900 (Japan) according to our previous studies (Duan et al., 2014).

### Determination of Serum Antioxidant Enzymes Activities and MDA Level

The activities of glutathione peroxidase (GSH-Px), catalase (CAT), and superoxide dismutase (SOD) and the level of malondialdehyde (MDA) in serum were measured with corresponding assay kits (Nanjing Bioengineering Institute, Nanjing, China) in accordance with the manufactures’ instructions. The GSH-Px activity was measured based on the principle that oxidation of glutathione (GSH) and hydrogen peroxide could be catalyzed by GSH-Px to produce oxidized glutathione (GSSG) and H_2_O. GSH can act with 5,5′-dithio-bis-2-nitrobenzoic acid to produce yellow 5-thio-2-nitrobenzoic acid. The change in absorbance during the conversion of GSH to GSSG was measured at 412 nm. The SOD activity was measured based on the auto-oxidation of hydroxylamine. The developed blue color was measured at 550 nm. CAT reacts with H_2_O_2_ and this reaction can be terminated by molybdenum to produce a yellow product. The activity of CAT was measured based on the decrease in absorbance at 405 nm due to the degradation of H_2_O_2_. The MDA level was measured based on the principle that MDA can react with thiobarbituric acid (TBA) to produce a red product (MDA-TBA), which was measured at 532 nm. The activities of GSH-Px, CAT, and SOD were expressed in U/mL, and the MDA level was shown in nmol/mL, respectively.

### Real-Time Quantitative (RT-PCR)

Total RNA from jejunum and ileum samples were isolated from liquid nitrogen using TRIZOL reagent (Invitrogen, United States) and then treated with DNase I (Invitrogen, United States) according to the manufacturer’s instructions. The reverse transcription was conducted at 37°C for 15 min, 85°C for 5 s. Primers used in this study were presented in the previous studies (Table 2). β-actin was chosen as the house-keeping gene to normalize target gene levels. Real-time PCR was performed according to our previous study (Yin et al., 2018). Relative expression was expressed as a ratio of the target gene to the control gene using the formula 2^–(ΔΔCt)^, where ΔΔCt = (Ct_*Target*_ – Ct_β –actin_) _*treatment*_ − (Ct_*Target*_ – Ct_β –actin_) _*control*_. Relative expression was normalized and expressed relative to the expression in the control group.

**TABLE 2:** Primers used for quantitative reverse transcription PCR.

Gene	Accession no.	Sequence (5′-3′)
β-actin	XM_0031242803	F: CTGCGGCATCCACGAAACT R: AGGGCCGTGATCTCCTTCTG
MnSOD	NM_001190422.1	F: GAGCTGAAGGGAGAGAAGACAGT R: GCACTGGTACAGCCTTGTGTAT
CuZnSOD	NM_214127.2	F: CTGGACAAATCTGAGCCCTAAC R: GACGGATACAGCGGTCAACT
GPx1	NM_214201	F: CGATGCCACTGCCCTCAT R: GGCCCACCAGGAACTTCTC
GPx4	NM_214407	F: GCTGGCTACAACGTCAAATTTG R: TCCCCTTGGGCTGGACTT
CAT1	NM_001012613	F: GGCAAGACCAAACTCTCCTTC R: AGCCTATCAGCATCCACACTG

*F, forward; R, reverse.*

### Gut Microbiota

Total genome DNA from colonic digesta was extracted using QIAamp DNA Stool Mini Kit and DNA concentration and purity was monitored on 1% agarose gels. The V3-V4 region of the bacterial 16S ribosomal RNA gene were amplified by using specific primer. Amplicons were extracted from 2% agarose gels and purified using the GeneJET gel extraction kit (Thermo Scientific) according to the manufacturer’s instructions. After quantified and purified, amplicons were sequenced. The sequences were analyzed and assigned to operational taxonomic units (OTUs; 97% identity). Alpha diversity was analyzed using QIIME (Version 1.7.0), which included calculation of ACE, Chao 1, Shannon, and Simpson indices. Beta diversity was analyzed using principal component analysis (PCA) and unweighted Unifrac cluster tree.

### Statistical Analysis

All data were analyzed using the one-way analysis of variance (ANOVA) followed by Duncan’s multiple comparisons (SPSS 22.0 software). In this study, comparisons were made between different experimental groups at the same time and no comparisons between different treatment times were made. Data are expressed as the mean ± SEM. Probability values ≤ 0.05 were taken to indicate statistical significance.

## Results

### D-Galactose Decreased Body Weight and Average Daily Feed Intake in Piglets

**FIGURE 1:**
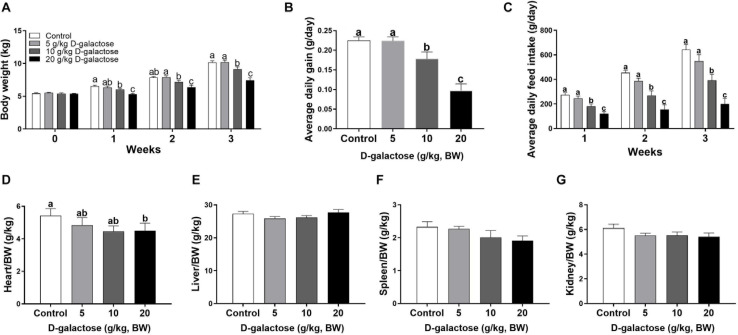
Effects of D-galactose on growth performance. **(A)** Body weight; **(B)** Average daily gain; **(C)** Average daily feed intake; **(D)** Relative weight of heart to body weight; **(E)** Relative weight of liver to body weight; **(F)** Relative weight of spleen to body weight; **(G)** Relative weight of kidney to body weight. Data were expressed as the mean ± SEM. Different lower-case letters indicate a significant difference among different dosage of D-galactose (*P* < 0.05).

To investigate whether D-galactose could induce chronic oxidative stress in piglets, we determined the growth performance and organ index of piglets fed the basal diet with different levels of D-galactose. The results showed that 10 and 20 g/kg BW D-galactose significantly reduced BW, ADG, and ADFI from the first week to the end as compared to the control and 5 g/kg BW D-galactose (*P* < 0.05) (Figures 1A–C). Notably, 10 g/kg BW D-galactose administration markedly decreased the heart/BW ratio at the third week (*P* < 0.05) (Figure 1D), while D-galactose failed to affect the liver/BW, spleen/BW, and kidney/BW ratio (*P* > 0.05) (Figures 1E–G).

### D-Galactose Had a Negative Effect Jejunum and Ileum Morphology in Piglets

**FIGURE 2:**
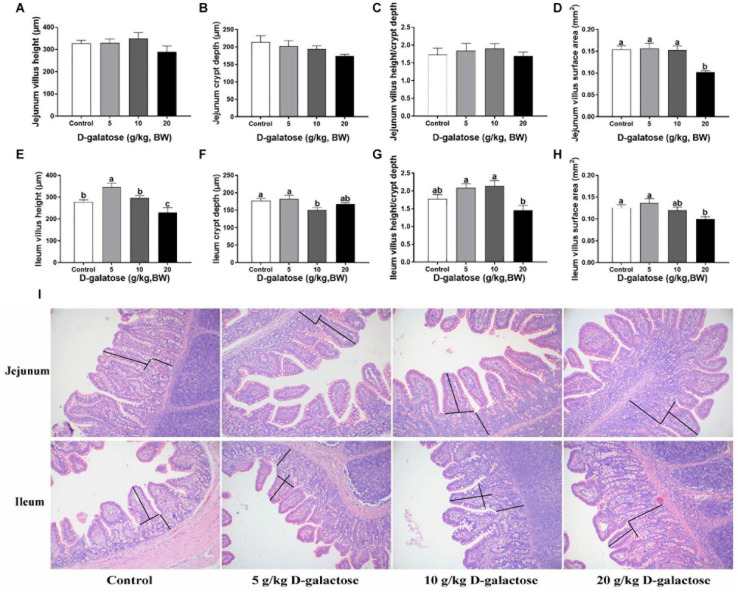
Effects of D-galactose on jejunal and ileal morphology. **(A)** Jejunum villus height; **(B)** Jejunum crypt depth; **(C)** Jejunum villus height/crypt depth; **(D)** Jejunum villus surface area; **(E)** Ileum villus height; **(F)** Ileum crypt depth; **(G)** Ileum villlus height/crypt depth; **(H)** Ileum villus surface area; **(I)** Representative images of HE staining in the jejunum and ileum. Data were expressed as the mean ± SEM. Different lower-case letters indicate a significant difference among different dosage of D-galactose (*P* < 0.05).

In the jejunum, although D-galactose have no significant effects on the villus height, crypt depth, and villus height/crypt depth (*P* > 0.05) (Figures 2A–C,I), the villus surface area of 20 g/kg BW D-galactose group was significantly lower than the other groups (*P* < 0.05) (Figure 2D). In the ileum, 5 g/kg BW D-galactose slightly increased villus height, whereas 20 g/kg BW D-galactose markedly reduced the villus height compared to the control group (*P* < 0.05) (Figure 2E). Meanwhile, 10 g/kg BW D-galactose decreased the crypt depth (*P* < 0.05) (Figure 2F). D-galactose had no significant effects on the villus height/crypt depth (*P* > 0.05) (Figures 2G,I). The villus surface area was significantly lower in the 20 g/kg BW D-galactose group than the control group (*P* < 0.05) (Figure 2H).

### D-Galactose Influenced Serum Biochemical Parameters

**FIGURE 3:**
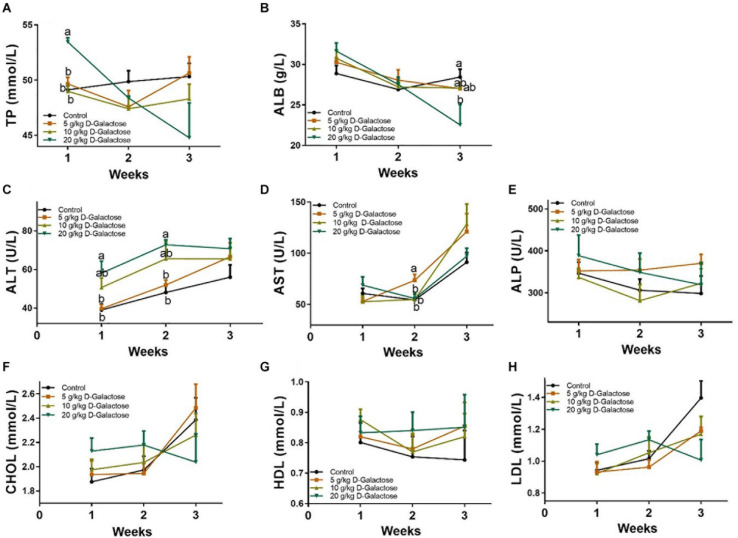
Effects of D-galactose on serum biochemical parameters. **(A)** TP; **(B)** ALB; **(C)** ALT; **(D)** AST; **(E)** ALP; **(F)** CHOL; **(G)** HDL; **(H)** LDL. Data were expressed as the mean ± SEM. Different lower-case letters indicate a significant difference among different dosage of D-galactose (*P* < 0.05).

At the first week, 20 g/kg BW D-galactose significantly increased the level of TP (*P* < 0.05) (Figure 3A), whereas 20 g/kg BW D-galactose markedly decreased the level of ALB at the third week (*P* < 0.05) (Figure 3B). The level of ALT was significantly higher in the 20 g/kg BW D-galactose group than that in the control and 5 g/kg BW D-galactose groups (*P* < 0.05) at the first 2 weeks (Figure 3C). 5 g/kg BW D-galactose markedly increased the level of AST compared to the other groups (*P* < 0.05) (Figure 3D). However, D-galactose had no effect on the levels of serum ALP, CHOL, HDL, and LDL in piglets (*P* > 0.05) (Figures 3E–H).

### D-Galactose Induced an Imbalance in Serum Amino Acids of Piglets

**FIGURE 4:**
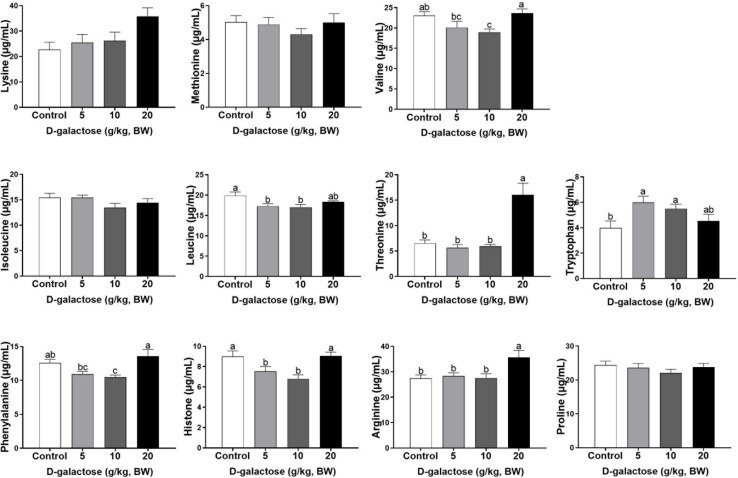
Effects of D-galactose on essential amino acids in the serum at the first week. Data were expressed as the mean ± SEM. Different lower-case letters indicate a significant difference among different dosage of D-galactose (*P* < 0.05).

**FIGURE 5:**
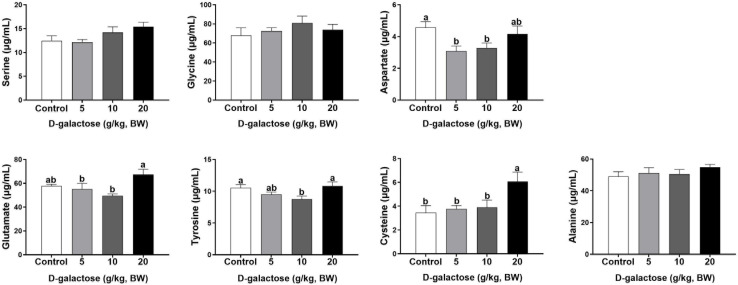
Effects of D-galactose on non-essential amino acids in the serum at the first week. Data were expressed as the mean ± SEM. Different lower-case letters indicate a significant difference among different dosage of D-galactose (*P* < 0.05).

At the first week, compared to the control group, 5 g/kg BW D-galactose significantly decreased the concentration of His, Leu, and Asp and increased the Trp content in serum (*P* < 0.05) (Figures 4, 5). The concentration of Val, Leu, His, Phe, and Tyr are significantly lower in 10 g/kg BW D-galactose group than that in the control group (*P* < 0.05) (Figures 4, 5). 20 g/kg BW D-galactose markedly increased the concentration of Arg and Thr in serum (*P* < 0.05) (Figure 4).

**FIGURE 6:**
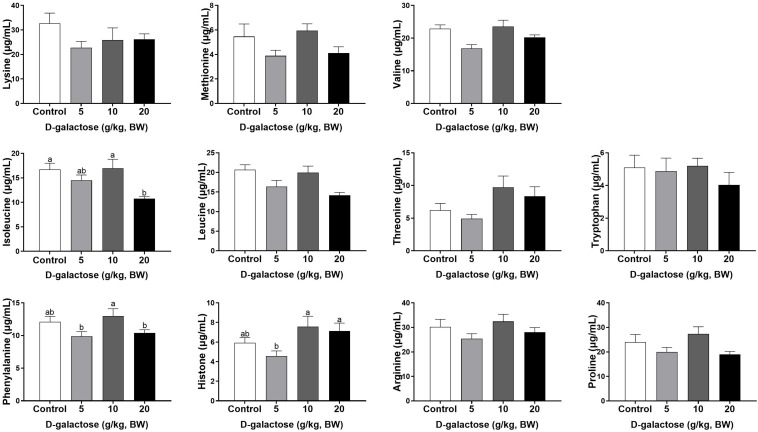
Effects of D-galactose on essential amino acids in the serum at the second week. Data were expressed as the mean ± SEM. Different lower-case letters indicate a significant difference among different dosage of D-galactose (*P* < 0.05).

**FIGURE 7:**
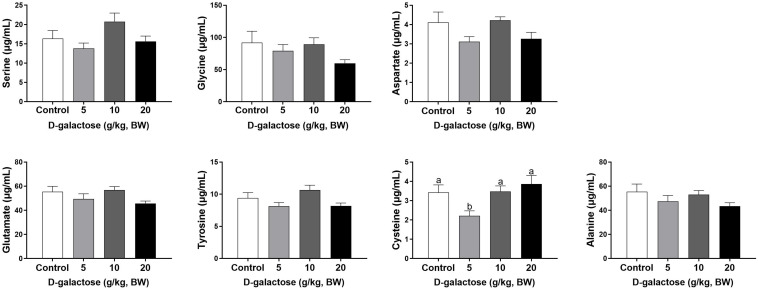
Effects of D-galactose on non-essential amino acids in the serum at the second week. Data were expressed as the mean ± SEM. Different lower-case letters indicate a significant difference among different dosage of D-galactose (*P* < 0.05).

At the second week, compared to the control group, 5 g/kg BW D-galactose significantly decreased the concentration of Cys, while 20 g/kg BW D-galactose significantly decreased the abundance of Ile (*P* < 0.05) (Figures 6, 7).

**FIGURE 8:**
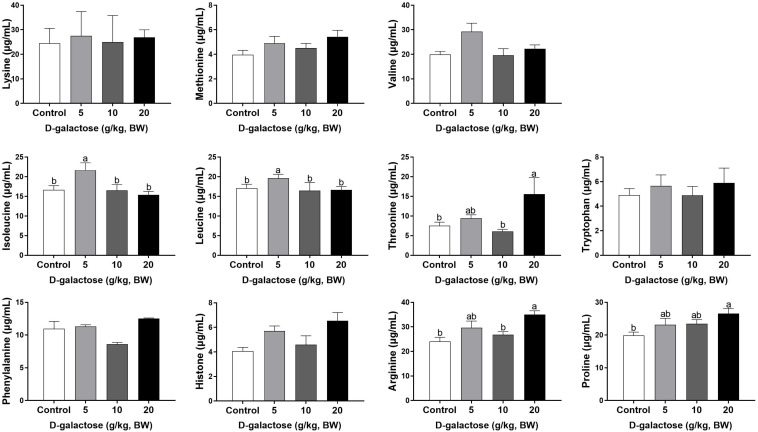
Effects of D-galactose on essential amino acids in the serum at the third week. Data were expressed as the mean ± SEM. Different lower-case letters indicate a significant difference among different dosage of D-galactose (*P* < 0.05).

**FIGURE 9:**
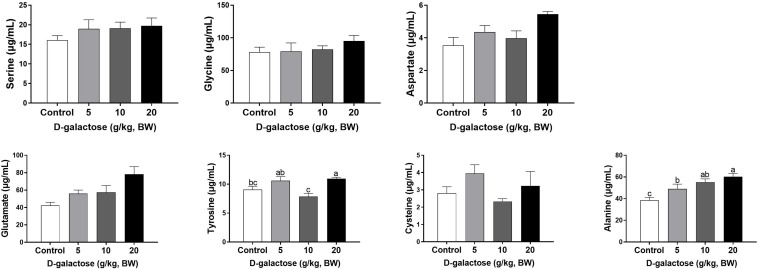
Effects of D-galactose on non-essential amino acids in the serum at the third week. Data were expressed as the mean ± SEM. Different lower-case letters indicate a significant difference among different dosage of D-galactose (*P* < 0.05).

At the third week, the concentration of Ile, Leu, and Ala in the 5 g/kg BW D-galactose group is significantly higher than that in the control group (*P* < 0.05) (Figures 8, 9). 10 g/kg BW D-galactose markedly increased the abundance of Ala and 20 g/kg BW D-galactose significantly increased the concentration of Arg, Thr, Pro, Ala, and Tyr in serum (*P* < 0.05) (Figures 8, 9).

### D-Galactose Affected Serum Antioxidant Enzyme Activities and MDA Level in Piglets

**FIGURE 10:**
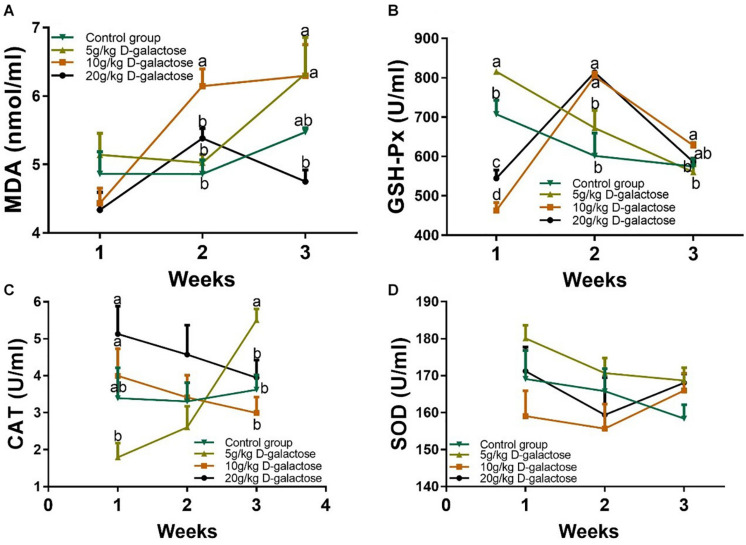
Effects of D-galactose on the level of MDA and activities of antioxidant enzymes. **(A)** MDA; **(B)** GSH-Px; **(C)** CAT; **(D)** SOD. Data were expressed as the mean ± SEM. Different lower-case letters indicate a significant difference among different dosage of D-galactose (*P* < 0.05).

To confirm whether the model of D-galactose induced piglet chronic oxidative stress was successful, serum antioxidant enzymes (GSH-Px, SOD, and CAT) activities and MDA level in piglets were measured. Although the MDA level at the first week were not changed among these groups, 10 g/kg BW D-galactose significantly increased the level of MDA (*P* < 0.05) at the second week (Figure 10A). 5 g/kg BW D-galactose significantly increased the level of MDA compared to the control and 20 g/kg BW D-galactose groups (*P* < 0.05) at the third week (Figure 10A). Meanwhile, 10 g/kg D-galactose (5.48 ± 0.06) tend to increase the MDA concentration compared to the group (6.29 ± 0.46) at the third week, although the difference was not significant (*P* > 0.05) (Figure 10A).

At the first week, the activity of GSH-Px markedly increased in response to 5 g/kg BW D-galactose, whereas 10 and 20 g/kg BW D-galactose significantly decrease the activity of GSH-Px (*P* < 0.05) (Figure 10B). However, 10 and 20 g/kg BW D-galactose significantly increased the activity of GSH-Px at the second week compared to the control and 5 g/kg BW D-galactose groups (*P* < 0.05) (Figure 10B). At the third week, 10 g/kg BW D-galactose markedly increased the activity of GSH-Px (*P* < 0.05) (Figure 10B). In addition, 5 g/kg BW D-galactose significantly reduced the activity of CAT when compared that of the 10 and 20 g/kg BW D-galactose groups (*P* < 0.05) at the first week (Figure 10C). 5 g/kg BW D-galactose markedly increased the activity of CAT compared to the other groups at the third week (*P* < 0.05) (Figure 10C). However, administration of D-galactose failed to affect the activity of SOD from the first week to the end (*P* > 0.05) (Figure 10D).

### D-Galactose Influenced the mRNA Expressions of GPx1, CAT1, and MnSOD in Jejunum and Ileum of Piglets

**FIGURE 11:**
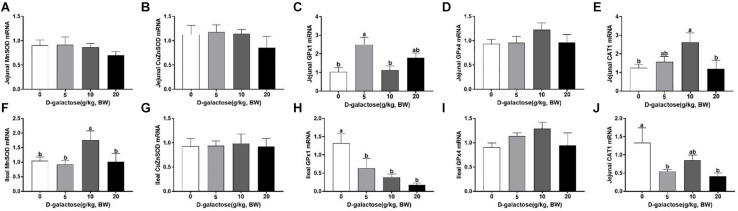
Effects of D-galactose on intestinal expressions of MnSOD, CuZnSOD, GPx1, GPx4, and CAT1. **(A–E)** mRNA abundances of MnSOD, CuZnSOD, GPx1, GPx4, and CAT1 in the jejunum; **(F–J)** mRNA abundances of MnSOD, CuZnSOD, GPx1, GPx4, and CAT1 in the ileum. Data were expressed as the mean ± SEN. Different lower-case letters indicate a significant difference among different dosage of d-galactose (*P* < 0.05).

D-galactose treatment had no significant effects on the jejunal mRNA expression of MnSOD, CuZnSOD, and Gpx4 (*p* > 0.05) (Figures 11A,B,D). 5 g/kg BW D-galactose significantly increased the jejunal mRNA expression of GPx1 compared with the control group (*P* < 0.05) (Figure 11C). 10 g/kg BW D-galactose increased the jejunal mRNA expression of *CAT1* (*P* < 0.05) (Figure 11E). In the ileum, the mRNA expression of *MnSOD* was significantly higher in the 10 g/kg BW D-galactose than that in the other groups (*P* < 0.05) (Figure 11F). Administration of D-galactose markedly reduced the expression of ileal *GPX1* (5,10, and 20 g/kg BW D-galactose) and *CAT1* (5 and 20 g/kg BW D-galactose) (*P* < 0.05) (Figures 11H,J). D-galactose failed to affect the mRNA expression of CuZnSOD and Gpx4 (*p* > 0.05) (Figures 11G,I).

### D-Galactose Influenced Colonic Microbiota in Piglets

The colonic microbiota was analyzed by sequencing V3 + V4 regions of 16S rRNA genes and an average of 72,951.48 raw reads were generated from each sample. After removing the low-quality sequences, an average of 68,692.55 clean tags were clustered into OTUs. To identify the microbial biodiversity, the reads were clustered into operation taxonomic units (OTUs) basing on 97% identity.

**FIGURE 12:**
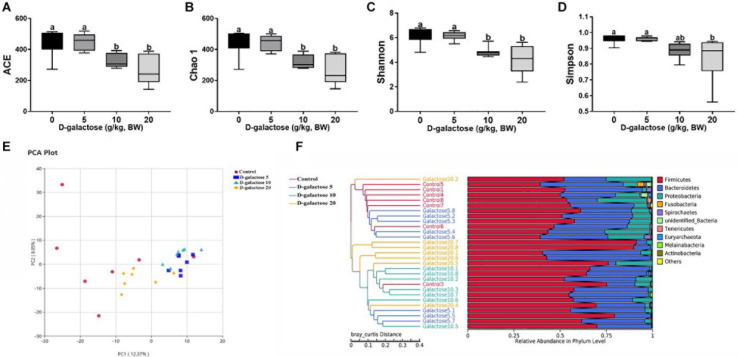
Effects of D-galactose on colonic microbial diversity and unweighted Unifrac distances. **(A)** ACE index; **(B)** Chao 1 index; **(C)** Shannon index; **(D)** Simpson index; **(E)** Principal Component Analysis (PCA); **(F)** Comparison of unweighted Unifrac distances between pairs os samples. Data were expressed as the mean ± SEN. Different lower-case letters indicate a significant difference among different dosage of D-galactose (*P* < 0.05).

ACE and Chao 1 indexes were examined for the community richness and Shannon and Simpson were examined for the community diversity. As shown in Figures 12A–C, compared to the control and 5 g/kg BW D-galactose groups, 10 and 20 g/kg BW D-galactose significantly decreased the ACE, Chao 1 and Shannon indexes (*P* < 0.05). Meanwhile, 20 g/kg BW D-galactose also significantly decreased the Simpson index when compared to the other groups (*P* < 0.05) (Figure 12D).

To further understand the microbial composition between different groups, we evaluated beta-diversity by using Principal Component Analysis (PCA) and unweighted Unifrac cluster tree based on UPGMA. The results showed that the microbial community structure in 10 and 20 g/kg BW D-galactose groups differed significantly from that in control and 5 g/kg BW D-galactose groups (Figures 12E,F).

**FIGURE 13:**
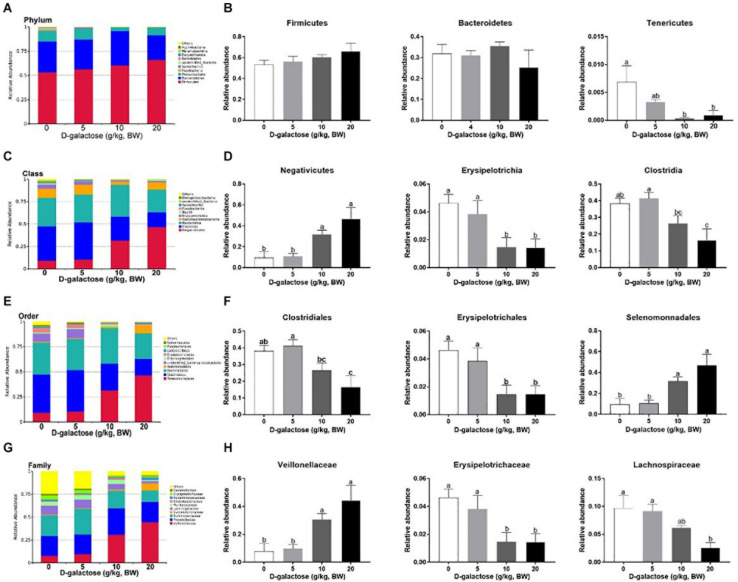
Effects of D-galactose on colonic macterial composition at the phylum, class, order, and family levels. **(A,B)** Phylum level; **(C,D)** Class level; **(E,F)** Order level; **(G,H)** Family level. Data were expressed as the mean ± SEN. Different lower-case letters indicate a significant difference among different dosage of D-galactose (*P* < 0.05).

The overall microbial composition in the groups differed at the phylum, class, order, and family levels. As shown in Figures 13A,B, at the phylum level, D-galactose (10 and 20 g/kg BW) significantly decreased the relative abundance of *Tenericutes* (*P* < 0.05), while it had little effect on the relative abundances of these two largest phyla (*Firmicutes* and *Bacteroidetes*) (*P* > 0.05). At the class level, compared to control and 5 g/kg BW D-galactose, 10 and 20 g/kg BW D-galactose significantly increased the relative abundance of *Negativicutes* and significantly decreased the relative abundance of *Erysipelotrichia* (*P* < 0.05) (Figures 13C,D). The relative abundance of *Clostridia* was significantly lower in 20 g/kg BW D-galactose groups than control and 5 g/kg BW D-galactose groups (*P* < 0.05) (Figures 13C,D). The order level analysis demonstrated that the relative abundance of *Clostridiales* was significantly lower in the 20 g/kg BW D-galactose group than the control and 5 g/kg BW D-galactose administration groups (*P* < 0.05) (Figures 13E,F). 10 and 20 g/kg BW D-galactose significantly decreased the relative abundance of *Erysipelotrichales*, while significantly increased the relative abundance of *Selenomonnadales* when compared to control and 5 g/kg BW D-galactose (*P* < 0.05) (Figures 13E,F). At the family level, 10 and 20 g/kg BW D-galactose significantly increased the relative abundance of *Veillonellaceae* and significantly decreased the relative abundance of *Erysipelotrichaceae* when compared to control and 5 g/kg BW D-galactose groups (*P* < 0.05) (Figures 13G,H). The relative abundance of *Lachnospiraceae* were significantly lower in the 20 g/kg BW D-galactose than the other groups (control and 5 g/kg BW D-galactose groups) (*P* < 0.05) (Figures 13G,H).

## Discussion

It has been reported that D-galactose could be changed into galactitol, which will accumulate in the cell and then generate the excess of ROS, thereby leading to oxidative stress (Zhang X. et al., 2019). Previous studies reported that administration of D-galactose could lower body weight and disrupt antioxidant enzymes in mice, thereby causing chronic oxidative stress (Mo et al., 2018; Zhang X. et al., 2019). Similarly, in the present study we found that 10 and 20 g/kg BW D-galactose decreased BW and ADFI from the first week to the end in piglets. The integrity of intestine is conducive to the absorption of various nutrients and form a protective barrier to defend kinds of stresses. It is known that oxidative stress can cause intestinal injury, such as decreasing villus height and increasing the production of ROS in the small intestine of piglets (Zheng et al., 2017; da Silva et al., 2019). In the present study, our results showed that addition of 20 g/kg BW D-galactose decreased villus height and villus surface area in ileum of piglets. In view of the above data, we speculated that excessive addition of D-galactose could affect the growth performance in piglets and disrupt ileal morphology. This may be that the accumulation of D-galactose was converted into indigestible galactitol, then which caused to generate amounts of ROS in the body, however, the detailed mechanisms need to be further studied.

Amino acids are essential nutrient which have been required for tissue protein synthesis and other metabolic function in animals. Our previous study has revealed that oxidative stress could cause alterations of serum amino acid levels in piglets (Duan et al., 2016). In this study, consistent with the decreased performance growth, D-galactose administration also changed the concentrations of serum amino acids, such as EAA His, Arg, Trp, Thr, Val, Leu, Phe, and NEAA Tyr, Cys, Asp. Amounting evidence has showed that amino acids involve in modulating oxidative stress. For example, Val and Leu can induce oxidative stress by producing excessive ROS (Zhenyukh et al., 2017), while Arg and Thr can ameliorate oxidative stress (Qiu et al., 2019; Yu et al., 2020). In this study, we found that10 g/kg BW D-galactose enhanced the serum concentration of Arg and Thr at the first week, which might protect host against D-galactose-induced oxidative stress; 5 and 10 g/kg BW D-galactose increased serum Val and Leu concentrations at the third week, which might aggravate oxidative stress. Serum ALP, AST, and ALT act as specific indicators of liver injury (Cao et al., 2019). Studies have showed that oxidative stress increased serum and liver AST and ALT levels in piglets (Di Giancamillo et al., 2015; Luo et al., 2016; Pu et al., 2016). Injection of D-galactose increased the serum levels of AST and ALT in mice, which indicated that D-galactose could cause liver injury (Lin et al., 2018). Hepatic AST is present in the cytoplasm and mitochondria and ALT is distributed in the cytoplasm (Feng et al., 2018). The damaged liver release AST and ALT into the circulation system, which enhanced the serum concentrations of AST and ALT (Feng et al., 2018). The present study found that 5 g/kg BW D-galactose significantly increased the concentration of serum AST at the second week. Moreover, 20 g/kg BW D-galactose significantly increased serum ALT level at the first 2 weeks, while decreased serum ALB level at the third week. However, D-galactose have no effect on the concentrations of serum ALP, AST, and ALT at the third week. These data suggested short-term exposure of D-galactose might lead to hepatic damage, while long-term exposure of D-galactose might have no harmful effect on liver in piglets due to feedback regulation.

Previous investigations have reported that D-galactose treatment decreased the activities of SOD, CAT, and GSH-Px and elevated the level of MDA in mice serum, liver, and brain (Qiu et al., 2017; Zhou et al., 2017; Lin et al., 2018; Ma et al., 2018; Zhang X. et al., 2019; Wang et al., 2020). However, D-galactose increased the activity of CAT and decreased the activity of SOD, while fail to affect the activity of GSH-Px in erythrocytes of rats (Delwing-de Lima et al., 2017). These results showed that D-galactose can induce oxidative stress and have various effects on different models. In this study, we also found that 5 g/kg BW D-galactose decreased the activity of CAT at the first week, while increased the activity of CAT at the third week. 10 g/kg BW D-galactose increased the activity of GSH-Px and the level of MDA at the second and third week. A study showed that antioxidant enzymes prevented hosts against oxidative stress by increasing their activities (Travacio and Llesuy, 1996). In addition, although it is still unclear that whether the oxidative stress is induced by weaning itself or other environmental factors, Zhu et al. (2012) found that weaning-induced oxidative stress decreased the activities of SOD and increased the level of MDA in serum of piglets (Zhu et al., 2012). Thus, we speculated that administration of D-galactose eventually could induce chronic oxidative stress in piglets *via* influencing the antioxidant enzymes activities.

Small intestine is the main organ for nutrient digestion and absorption in mammals and is highly susceptible to oxidative stress (Duan et al., 2014; Yin et al., 2015; Hussain et al., 2016). To further investigate whether D-galactose could induce chronic oxidative stress, the mRNA expressions of antioxidative related genes GPx1, CAT1, and MnSOD in jejunum and ileum were determined. In this current study, we found that 5 and 10 g/kg BW D-galactose increased jejunal GPx1and CAT1 expressions, respectively. 5, 10, and 20 g/kg BW D-galactose downregulated ileal GPx1 and CAT1 expression in piglets, which is consistent with the previous study (Mo et al., 2018). Evidence has showed that oxidative stress increased the mRNA expression of GPx1 in the jejunum of piglets (Cheng et al., 2019). Similarly, an *in vitro* study also showed that D-galactose decreased the mRNA expressions of CuZnSOD, MnSOD, and GPx4 in IPEC-J2 (Cao et al., 2020). These data showed that D-galactose could induce intestinal oxidative stress by modulating the expressions of antioxidant enzymes. Our previous study also found that essential nutrient which hav oxidative stress enhanced the mRNA expressions of CuZnSOD and GPx1 in the jejunum and ileum of piglets (He et al., 2018). Therefore, we speculated that in the jejunum, 5 and 10 g/kg BW D-galactose might exhibit a feedback regulatory mechanism against oxidative stress *via* enhancing GPx1 and CAT1 expression. However, in the ileum, D-galactose treatment inhibited antioxidant capacity since it decreased the expressions of GPx1 and CAT1. Additionally, we speculated that D-galactose might only induce alteration of antioxidant status in the ileum in piglets.

Additionally, amounting studies indicated that oxidative stress has direct correlation with gut microbiota in piglets and sows (Qiao et al., 2013; Li et al., 2019; Nie et al., 2019; Wang et al., 2019). A previous study has showed that *Tenericutes* is associated with improving apparent crude fiber digestibility in pigs (Niu et al., 2015). In addition to this, the relative abundance of Tenericutes is higher in healthy humans than individuals with metabolic disorders (Lim et al., 2017). Oxidative stress decreased the relative abundance of Tenericutes in rats (Zhou et al., 2018). Consistently, in this study, we also found that 10 and 20 g/kg BW D-galactose downregulated the relative abundance of *Tenericutes*. These results suggested that D-galactose supplementation had negative effect on metabolism in piglets. *Lactobacillus frumenti* appears to improve the antioxidant capacity in weaned piglets (Nie et al., 2019). Studies have showed that *Lactobacillus and Bifidobacterium* can alleviate oxidative stress *via* scavenging free radicals (Lin and Chang, 2000). However, in the current study, D-galactose supplementation had no effect on the relative abundance of *Lactobacillus* and *Bifidobacterium.* A study have showed that oxidative stress increased the relative abundance of *Clostridium perfringens*, *E. coil*, and *enterococcus*, while decreased the relative abundance of *lactobacilli* in mice (Qiao et al., 2013). Decreased relative abundance of *Erysipelotrichia* and *Clostridia Chronic* have been reported to contribute to gastrointestinal disease (Minamoto et al., 2015). In addition, injection of D-galactose increased the relative abundance of *Firmicutes* and *Clostridiales*, while decreased the relative abundance of Bacteroidetes and *Lactobacillus* in mice (Zhao et al., 2018). In the current study, D-galactose treatment decreased the relative abundance of *Erysipelotrichia* and *Clostridia* classes and *Clostridial*es and *Erysipelotrichales* orders. Based on these results, we infer that D-galactose could reduce microbial diversity and further cause metabolic dysbiosis and intestinal diseases in piglets.

In conclusion, we suggested that the optimal modeling method is a final dosage of 10 g/kg BW/day D-galactose treated with weaned piglets for 3 weeks for D-galactose-induced chronic oxidative stress modeling. Additionally, administration of D-galactose had negative effects on the growth performance and influenced gut microbiota in piglets.

## Data Availability Statement

The datasets presented in this study can be found in online repositories. The names of the repository/repositories and accession number(s) can be found in the article/supplementary material.

## Ethics Statement

The animal study was reviewed and approved by Institute of Subtropical Agriculture, Chinese Academy of Sciences (2013020; Changsha, China). Written informed consent was obtained from the owners for the participation of their animals in this study.

## Author Contributions

HH, ZL, and JY conducted the study. JG, RH, and CW helped to perform the experiment. LH, XH, GW, and TL helped to writing the manuscript. TL and YY designed the experiment and revised the manuscript. All authors contributed to the article and approved the submitted version.

## Conflict of Interest

XH and GW are employed by the company Changsha Lvye Bio-Technology Co., Ltd. The remaining authors declare that the research was conducted in the absence of any commercial or financial relationships that could be construed as a potential conflict of interest.
